# Land Use, Climate, and Water Resources—Global Stages of Interaction

**DOI:** 10.3390/w9100815

**Published:** 2017-10-24

**Authors:** Sujay S. Kaushal, Arthur J. Gold, Paul M. Mayer

**Affiliations:** 1 Department of Geology & Earth System Science Interdisciplinary Center, University of Maryland, College Park, MD 20740, USA; 2 Department of Natural Resources Science, University of Rhode Island, Kingston, RI 02881, USA; 3 National Health and Environmental Effects Research Lab, Western Ecology Division,US Environmental Protection Agency, 200 SW 35th Street, Corvallis, OR 97333, USA

**Keywords:** land use, climate, urbanization, agriculture, dams, stream burial, urban evolution, salinization, Anthropocene, stream restoration, best management practices, models

## Abstract

Land use and climate change can accelerate the depletion of freshwater resources that support humans and ecosystem services on a global scale. Here, we briefly review studies from around the world, and highlight those in this special issue. We identify stages that characterize increasing interaction between land use and climate change. During the first stage, hydrologic modifications and the built environment amplify overland flow via processes associated with runoff-dominated ecosystems (e.g., soil compaction, impervious surface cover, drainage, and channelization). During the second stage, changes in water storage impact the capacity of ecosystems to buffer extremes in water quantity and quality (e.g., either losses in snowpack, wetlands, and groundwater recharge or gains in water and nutrient storage behind dams in reservoirs). During the third stage, extremes in water quantity and quality contribute to losses in ecosystem services and water security (e.g., clean drinking water, flood mitigation, and habitat availability). During the final stage, management and restoration strategies attempt to regain lost ecosystem structure, function, and services but need to adapt to climate change. By anticipating the increasing interaction between land use and climate change, intervention points can be identified, and management strategies can be adjusted to improve outcomes for realistic expectations. Overall, global water security cannot be adequately restored without considering an increasing interaction between land use and climate change across progressive stages and our ever-increasing human domination of the water cycle from degradation to ecosystem restoration.

## 1. The Emerging Global Water Crisis

Over 75% of Earth’s land surface has now been impacted by human development [1], which exerts an expanding footprint on water resources. There are distinct patterns in the evolution of land use change [2,3], and land use is a template on which climate interacts to influence the quantity and quality of Earth’s water [4]. In order to support human-dominated land use, ground water in semi-arid and arid landscapes has been decreasing by approximately 150 cubic km per year due to increasing extraction [5] (and references there in). Water stored as ice on land, which is critical for major drinking water supplies, has decreased at a rate of approximately 300 cubic km per year due to warming and changes in regional precipitation patterns [5] (and references there in). In fact, access to clean water is considered one of the contemporary grand challenges for engineering by the U.S. National Academy of Engineering (http://www.engineeringchallenges.org/challenges/water.aspx). Globally, major impacts are being caused by the increasing irrigation and accompanying dam construction and groundwater extraction [6]. Thus, a water crisis has been proposed as a major global issue [7,8], and much work has described impairments and alterations of water systems [9,10]. However, critical questions remain regarding our current state of the world’s water and how water impairments have evolved over time [11]. Can we alter our future course to avoid a global water crisis and related pitfalls [7]? How will we manage water in the future [12,13]. The papers in this special issue on Land Use, Climate, and Water Resources provide practical information and, along with case studies from different regions of the world, help address some of the most important water management questions facing us now.

## 2. Human Domination of Water-from Degradation to Restoration Cycles

**Figure 1:**
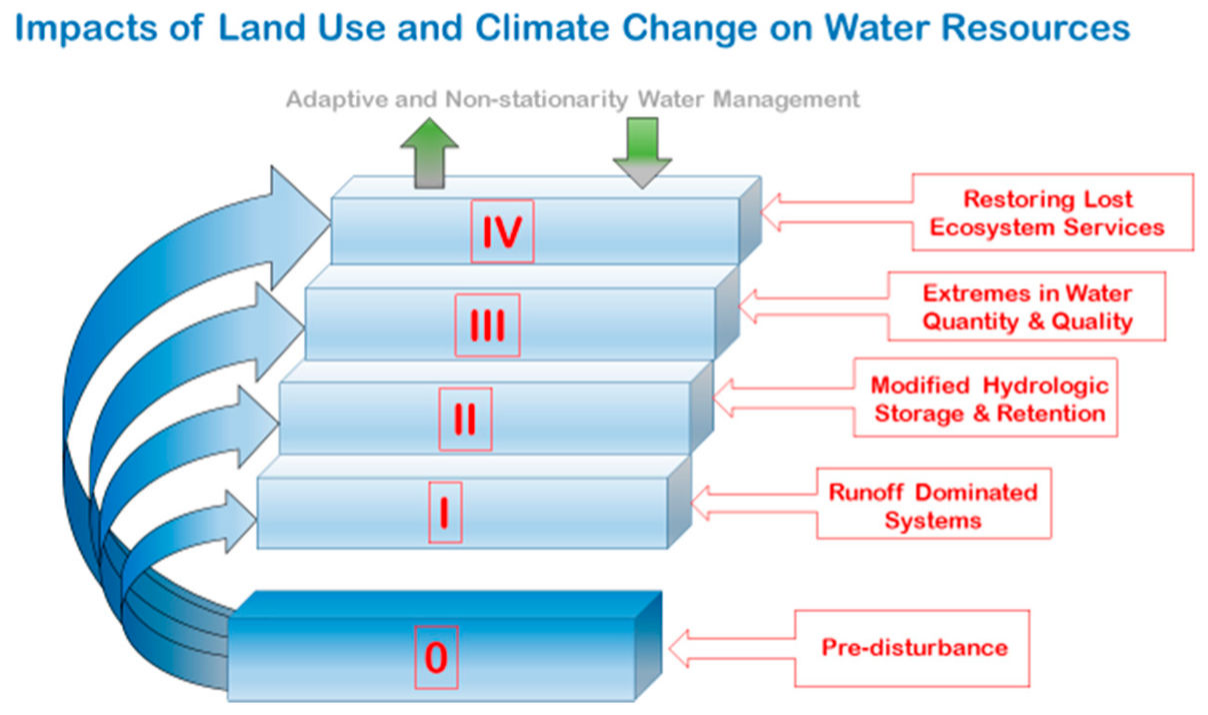
Conceptual model describing how land use and climate change alter the amount and quality of water across each step of human domination of Earth’s water cycle from degradation to ecosystem restoration.

Humans have dominated Earth’s ecosystems over millennia [14]. Since recorded history, humans have influenced every major hydrologic process of the water cycle including: altered rainfall regimes (e.g., modified by urban areas), accelerated runoff and overland flow from impervious surfaces and tile drains, reduced infiltration from soil compaction and impervious surfaces [15], and induced changes in evapotranspiration from irrigated agriculture and trees in urban areas. Recharge has also changed due to consumptive processes in ground water, and there is also less recharge due to melting snowpacks and shifts in regional rainfall patterns [16]. Water systems also evolve over time due to adaptations in response to selective pressures and decisions by humans [2,3]. Furthermore, there are coinciding changes in aquatic ecosystem structure, function, and associated services, coupled with vulnerability to climate at each step of human domination of the water cycle from degradation to restoration (Figure 1). It is now well known that human activities have contributed to increasing emissions of greenhouse gases, which is responsible for regional climate change and variability—this can spur regional and local adaptation and mitigation strategies [17,18]. However, restoration efforts to regain losses in structure, function, and services are unrealistic unless they consider the path of degradation of water systems (Figure 1). Thus, restoration and water management are “ex post facto” responses with an “impair and then repair” approach [11]. Instead, we need a predictive and proactive approach based on an understanding of both root causes and trends of water degradation and an appreciation for nonstationarity and uncertainty in climate trends—e.g., historical trends in the past do not always help us predict future changes in water management [12]. Overall, the losses in structure, function, and services due to land use change have contributed to a global water crisis related to the resiliency and resistance of water to climate change.

Although there is interest in ecosystem restoration globally, there is widespread recognition that it is not possible to return to historical pre-disturbance conditions prior to human activities. As land use change proceeds globally, it is inevitable that water systems and their associated ecosystem services will progress through a series of stages. Tracking the evolution of the global water crisis can not only help diagnose a global syndrome impairing water quantity and quality, e.g., [10], but it can also inform realistic management and ecosystem restoration. Specifically, it can help to (1) evaluate the severity of impairments across stages, (2) anticipate and/or predict the prognosis of water quantity and quality over time, (3) improve and inform monitoring of water quantity and quality over time, (4) identify and detect stability, resistance, and resilience of water resources over time, and (5) select appropriate infrastructure management and/or ecosystem restoration interventions over the course of time.

## 3. Characterizing Interactive Stages of Land Use and Climate Change

During the present geological epoch of the Anthropocene, pollution from agriculture and urbanization have increased globally at the same time that warming trends and climate extremes have increased in frequency and intensity [17,19,20]. Additionally, regional issues related to water consumption and droughts and floods further limit water sustainability. Below, we review papers from the special issue, characterizing the interactive stages of land use and climate change on water resources. Collectively, these papers explore: (1) human alteration of the ecosystem structure and function of headwaters and drainage networks; (2) alterations in hydrologic storage and biogeochemical retention in ground water, streams, and wetlands (e.g., reduced snowpack, infiltration, and recharge, etc.); (3) changes in ecosystem services of water in response to land use and climate change; and (4) changes in watershed management, adaptation, and restoration strategies to offset increasing effects of land use and climate change.

### 3.1. Stage 1: Hydrologic Modifications and the Built Environment Amplify Water Losses via Runoff-Dominated Systems

Increases in runoff can be driven by complex vegetation dynamics that differ from region to region. For example, studies have shown that in North American regions (e.g., Canada), elevated temperatures have led to lower temperature stress on vegetation [21], which can contribute to denser vegetation in response to higher CO_2_ levels [22], thereby reducing runoff coefficients [23]. However, in other regions, higher temperatures can lead to a declines in vegetation due to stress on plant communities, which can actually increase runoff [21]. Thus, there may be positive or negative effects on runoff based on regional land cover and vegetation dynamics.

Given these complexities regarding land cover and vegetation dynamics, we focus primarily on the interaction between agricultural and urban drainage infrastructure and climate variability. Engineered drainage of landscapes and soil compaction are widespread hydrologic modifications leading to run-off dominated systems during storm flow [4]. This is compounded by changes in the frequency and intensity of regional precipitation patterns due to global climate change [18] and changes in the characteristics of soils through land development. For example, the constant working of the land to produce a crop often erodes and compacts the soil and changes its structure, sometimes making it more difficult for water to seep into the ground, move through the soil, and mix with groundwater [24,25]. In addition, humans have used drainage ditches and pipes to bury headwaters and alter the structure and function of river networks as a whole [26]. Streams and rivers have become more efficient at conveying runoff especially due to channelization [27]. In this special issue, Weitzell et al. quantify the spatial extent of stream burial in the mid-Atlantic USA [28]. They found that patterns of stream burial are significantly related to (1) the watershed area and the size of streams; (2) the topography of the drainage area; and (3) the watershed impervious surface area. Interestingly, they found that patterns of local stream burial have changed over time in response to evolving zoning plans and land use management. They point out that mapping patterns of stream burial and changes over time can be used to evaluate how hydrologic connectivity is expanding at regional scales, and, consequently, there is greater potential for greater conveyance of pollutants from uplands to streams during storms.

The effects of the artificially expanded drainage networks on stream discharge and water chemistry are still poorly understood [29]. Previous work has shown that stream burial can reduce nitrogen uptake and contribute to increased transport of carbon and nitrogen in streams and river networks [30,31]. In this special issue, Gannon et al. point out that ephemeral drainage ditches draining roads are excluded from the jurisdiction of the Clean Water Act [32]. They show that even though these ditches represent relatively small areas of the watershed, they magnify runoff levels significantly when compared to larger streams. They also found that these drainage ditches can alter the calcium, silicon, and sulfate chemistry of runoff. Recent work shows that concentrations of these major ions are dramatically increased across developed land use due to human-accelerated weathering and anthropogenic salt inputs [33]. Given that these ditches are common in many landscapes globally, there is potential to amplify degraded water quality pulses in response to extreme events at a regional scale in response to climate variability.

### 3.2. Stage 2: Losses in Water Storage and Ecosystem Retention Reduce the Capacity of Ecosystems to Buffer Extremes in Water Quantity and Quality

Land use and climate change can alter water storage and ecosystem retention [15]. Deforestation can decrease water storage and retention by vegetation. Increases in urban impervious surface cover and artificial drainage can also reduce infiltration and groundwater storage. As urbanization expands, cities require more water and can increase groundwater withdrawal from aquifers faster than recharge, which reduces storage. In mountainous regions, decreased storage in melting snowpacks also contributes to increased runoff and nitrogen loading to streams [34]. In this special issue, Yin et al. show that the water balance has also changed in mountainous watersheds in China, with an increase in runoff by 30.5% from 1964 to 2013 [35]. Changes in climate and groundwater flow have contributed to increasing runoff and decreased water storage in this region of China, which can influence uses for drinking water and agriculture. In this special issue, Johannsen et al. also explored the future of shifting water supply and water demand with serious implications for the sustainability of societies in the Middle East and North Africa [36]. They project a significant decrease in available water resources in Morocco up to 2029 and predict an increase in water demand. The decrease in water supply is due to decreased storage in the aquifer caused by increasing use for irrigation, particularly during periods of water stress under drought conditions.

Globally, there has been a shift from groundwater recharge to artificial storage of surface waters behind dams [15]. The industrial revolution at the beginning of the 19th century is a seminal moment in the Anthropocene water management, where connectivity and flow regimes underwent rapid alterations, damaging fish passage, and changing sediment dynamics [37]. Dams also have contributed to accelerated alteration of semi-arid and arid lands at the start of the 20th century, which spawned high production agriculture in the Western U.S. This has generated substantial food and fiber, but strongly altered water flows and quality [38]. In this special issue, Gold et al. in a study of thousands of dams in New England show that, while some dams with reservoirs can serve to reduce nitrogen export to coastal waters, in most cases, dam removal to improve flow regimes, fish passage, or hazard reduction will not substantially increase watershed N export [39].

### 3.3. Stage 3: Extremes in Water Quantity and/or Quality Lead to Local Losses in Ecosystem Services and Regional Water Security

In this special issue, Awal et al. predicts that climate extremes will increase variability and precipitation in the headwaters of the Brazos River in Texas, USA [40]. The growing economy and population will increase water demand during periods of water stress. Similarly, the paper by Gu et al. in this issue analyzes changes in stream flow and the relationship with climate variation and anthropogenic activities in the Poyang Lake basin in China [41]. Poyang Lake is the largest freshwater lake in China and is connected to the Yangtze River. Gu et al. found that reservoir construction largely altered annual distribution of stream flow. While there was a shift towards runoff-dominated ecosystems, artificial storage and retention by dams can play a major role in buffering the effects of land use and climate change in watershed (as discussed previously). Finally, the paper by Kamarinas et al. in this special issue investigated the water quality responses of intensively managed landscapes to precipitation events with a focus on sediment dynamics [42]. They found that different types of land use related to deforestation and afforestation showed different patterns of total suspended solids in water clarity. Thus, there may be important legacy effects of land-use, which influence water quality over relatively longer time periods following disturbance.

### 3.4. Stage 4: Water Management and Restoration Strategies Aim to Regain Losses in Ecosystem Structure, Function, and Services

However, there is growing interest and effort in restoration and management of watersheds to mitigate the effects of land use and climate change, there can be unanticipated trade-offs and unintended consequences that alter water quality. For example, while previous work has shown that storm water management control structures and stream restoration can retain nitrogen due to denitrification [19,43,44], other work shows that phosphorus can be released from sediments in response to decreased oxygen availability [45]. In the special issue, Duan et al. show that phosphorus can be released from sediments in storm water management control structures during low flow due to changes in redox potential, warmer temperatures, and internal loading from sediments to streams [45]. Duan et al. suggest that planting macrophytes and/or dredging and removing sediments can potentially increase the effectiveness of storm water management controls to retain nitrogen and phosphorus.

Stream restoration strategies may have the capacity to retain nitrogen, but efficiency may be influenced by environmental variables. In the special issue, Newcomer Johnson et al. conduct a global review of stream restoration strategies to reduce nation loads to rivers [46]. They showed statistically significant relationships between nutrient uptake and stream and watershed attributes such as watershed size, impervious surface cover, discharge, and other environmental variables depending on the form of nitrogen and phosphorus. They also developed a typology characterizing different forms of stream and river restoration which can be used to identify the most effective mitigation strategies for the interactive effects of land use and climate change based on the likely response of management across the range of restoration characteristics. Thus, the most appropriate, watershed-based strategies can be implemented for reducing nutrient exports and managing hydrologic residence times and hydrologic connectivity using storm water management and conservation approaches.

## 4. The Future of Water: Stepping into the Unknown

Over time, human actions change hydrology and water movement through ecosystems. Management may be necessary to address excess water, e.g., storm water runoff, or water scarcity and decreased water quality e.g., access to clean drinking water. In addition, extremes in climate are increasing regionally and there is nonstationarity in climate and water [12]. Regions that currently have favorable hydrology may not in the future. Regions that do not have favorable hydrology must adapt by investing in infrastructure to manage and store water. As illustrated in this special issue, fresh water is not distributed equally globally, with many regions receiving too little or too much water, and this will impact regional differences in stages of the global water crisis.

There are many research frontiers and unknowns related to the interactive effects of land use and climate on water. We focus on four specific research needs related to intersections between (1) hydrogeology and land use, (2) remote sensing and hydrology (3) ecology and engineering, and (4) translating watershed research into management. First of all, land use has a vulnerability to climate based on geologic setting and topography. Geologic setting is important to water quantity and quality because of its fundamental influence on water movement, infiltration, erosion rates, and chemistry. However, it is still underappreciated that urbanization creates its own distinct geology, which can dramatically influence the transport and chemistry of water in the built environment [2,3]. Stormdrain and ditch networks dramatically alter drainage networks and geologic materials in close contact with water [28,32]. These drainage structures and geologic materials significantly affect water chemistry [32]. Land use change can alter geological processes such as human-accelerated weathering of impervious surfaces, which can increase many major ions in streams and rivers [33]. Thus, a research frontier is elucidating the impacts of impervious surfaces (as geologic materials subject to degradation and weathering) on the chemistry and quality of fresh water.

Secondly, remote sensing can provide new information related to land use and climate change and corresponding feedbacks on changes in hydrologic processes that would be difficult or impossible to quantify [47]. For example, we can now characterize changes in land use and land cover and conversion to agricultural lands, which can influence water recycling rates [48] evapotranspiration, and runoff processes [48,49]. High-resolution satellite date can allow us to determine finer scale variations in the energy balance of human-dominated watersheds as well as changes in regional water balances due to land use, land cover, and management decisions [50]. This is important in urbanizing regions given the expanding impacts of urban heat islands [51,52], and their contribution along with climate change on rising river temperatures and related impacts on water quantity and quality [24,53].

Thirdly, there is often a disconnection between ecology and engineering in managing water resources, although access to clean water is a grand challenge for engineering, as mentioned earlier. Government agencies sometimes seek engineering solutions as opposed to ecological ones, likely because ecology has many complexities, uncertainties, and it takes time to collect enough monitoring data to comprehend all the interactions in an abiotic and biotic system. Engineering solutions are often sought to meet a specific regulation and permit without fully considering ecological interactions. As studies in this special issue point out, there are many interactive effects associated with land use and climate change, which should be considered. For example, the interactive effects of urbanization and climate variability can be managed by regenerative stormwater conveyance in some urban areas [54]. Regenerative stormwater conveyance is an example of an engineering approach that can enhance denitrification by creating favorable redox conditions. Yet, regenerative stormwater conveyance can also trigger release of iron during these redox conditions, which is an unintended consequence and can impact aquatic habitat by creating dense mats of iron flocculate [54]. Sometimes engineering solutions are implemented without a full understanding of the ecosystem consequences. As an initial step, ecohydrological modeling can be used to better incorporate ecology and biogeochemistry into engineering solutions [55]. These models can use easily accessible environmental data that was not available in the past such as soil moisture, temperature, topography, irradiance, and remotely sensed data that can be used as model inputs. Integrated modeling encompasses multiple processes [56] and preferably uses standard interfaces [57]. Data related to environmental monitoring is not always readily available, and there is a need for automated sampling stations, as highlighted by previous work [58]; this can pose a problem in integrated eco-hydrological modeling studies [24,59]. Ultimately, monitoring is still critical for optimizing better ecological and engineering solutions and detecting unintended consequences that cannot be easily anticipated (please see below).

Fourthly, translating watershed research into management also remains a frontier for the future of water resources [60]. In many instances, regulations cannot keep pace with the science—there can be a lag time between dissemination of monitoring results and the development and implementation of management and policy. Watershed managers, stakeholders, and decision-makers are often left with uncertainties with which strategies or technologies they should pursue or how they should address an emerging problem in water resources management [61]. Thus, science and technology transfer often falls short because it is either difficult to communicate messages and/or there needs to be strong incentives for scientists and managers to work together [62]. In some cases, one scientist may have a disproportionate influence on policymakers due to their communication approaches, personality, style, and/or marketing of research, where different viewpoints may be overlooked. One way to integrate multiple viewpoints is through the establishment of expert panels, which can bring together broader groups of watershed managers and scientists to influence the evolution of water and biogeochemical cycles [2]. Scientific and engineering information can then be synthesized by communications specialists via webinars, workshops, technical reports, interactive websites, and training programs.

Across the world, the studies in this special issue and growing work suggest that future trends in water management will not be static in the future [63] (Figure 1). Instead, they will need to be dynamic processes based on adaptive management. Thus, we will need to keep evaluating whether management approaches are still effective in response to the increasing interaction between land use and climate change on a global stage. This brief review and studies in this special issue suggest that conservation of natural lands (in addition to *ex post facto* restoration) is critical to slow down and/or reverse the interactive effects of land use and climate on water resources [64]. Overall, global water security cannot be adequately restored without considering an increasing interaction between land use and climate change across progressive stages and our ever-increasing human domination of the water cycle from degradation to ecosystem restoration.
